# FungiExpresZ: an intuitive package for fungal gene expression data analysis, visualization and discovery

**DOI:** 10.1093/bib/bbad051

**Published:** 2023-02-17

**Authors:** Chirag Parsania, Ruiwen Chen, Pooja Sethiya, Zhengqiang Miao, Liguo Dong, Koon Ho Wong

**Affiliations:** Faculty of Health Sciences, University of Macau, Macau SAR of China; Faculty of Health Sciences, University of Macau, Macau SAR of China; Faculty of Health Sciences, University of Macau, Macau SAR of China; Faculty of Health Sciences, University of Macau, Macau SAR of China; Faculty of Health Sciences, University of Macau, Macau SAR of China; Faculty of Health Sciences, University of Macau, Macau SAR of China; Institute of Translational Medicine, University of Macau, Macau SAR of China

**Keywords:** FungiExpresZ, bioinformatics tool, RNA-seq database, data visualization, data analysis, fungi

## Abstract

Bioinformatics analysis and visualization of high-throughput gene expression data require extensive computer programming skills, posing a bottleneck for many wet-lab scientists. In this work, we present an intuitive user-friendly platform for gene expression data analysis and visualization called FungiExpresZ. FungiExpresZ aims to help wet-lab scientists with little to no knowledge of computer programming to become self-reliant in bioinformatics analysis and generating publication-ready figures. The platform contains many commonly used data analysis tools and an extensive collection of pre-processed public ribonucleic acid sequencing (RNA-seq) datasets of many fungal species, including important human, plant and insect pathogens. Users may analyse their data alone or in combination with public RNA-seq data for an integrated analysis. The FungiExpresZ platform helps wet-lab scientists to overcome their limitations in genomics data analysis and can be applied to analyse data of any organism. FungiExpresZ is available as an online web-based tool (https://cparsania.shinyapps.io/FungiExpresZ/) and an offline R-Shiny package (https://github.com/cparsania/FungiExpresZ).

## Introduction

Next-generation sequencing (NGS) costs have reduced dramatically in recent years [1, 2], rendering NGS experiments highly affordable. Ribonucleic acid sequencing (RNA-seq) has now become a standard research tool, with colossal amounts of sequencing data deposited in public databases such as Sequence Read Archive (SRA) or Gene Expression Omnibus. Integration of these freely available data has vast potential for addressing questions that would not be possible by individual studies.

Transcriptome profiles and genomics data are complex, requiring bioinformatics programming skills (e.g. R, Python and/or Bash) for data processing, visualization and downstream analysis. It has been estimated that more than 50% of wet-lab scientists lack these basic bioinformatics skills [3]. Various software tools have been developed to support wet-lab scientists with their data analysis. For instance, Digital Expression Explorer [4] computes normalized gene expression values from raw sequencing data. Multiple web-based tools allow differential expression analysis and visualization of user-supplied gene expression data. Some representative tools include MetaOmics [5], degust [6], START [7], o-miner [8], iDEP [9], IRIS-EDA [10], ExpressViz [11] and eVITTA [12]. Galaxy, a widely-used cloud-based bioinformatics workbench, provides convenient access to many commonly used analysis tools [13]. However, the standard analysis pipeline of data processing, data visualization and generation of publication-ready figures involves switching between multiple tools on the platform, posing a technical challenge for novice bioinformaticians and wet-lab scientists. Other bioinformatics packages integrating multiple data analysis tools are proprietary and only commercially available (e.g. GenomeStudio, Ingenuity Pathway Analysis and Dr Tom). Community-specific databases such as FungiDB [14] store gene expression data and provide pre-configured workflows for gene expression data analysis. However, data visualization options and public data availability are limited. Therefore, many wet-lab scientists (and even some bioinformaticians) cannot fully exploit the valuable and rich information offered by NGS applications and the vast amounts of freely available public NGS data.

In this study, we developed an intuitive user-friendly platform called FungiExpresZ (**Fung**al Gene **Expres**sion Data Analysis and Visuali**Z**ations) to facilitate data analysis and generation of publication-ready graphs. This platform can generate 19 different types of commonly used graphs and provide routines for standard bioinformatics tasks such as functional enrichment analysis, principal component analysis (PCA) and clustering analysis. At the time of publication, FungiExpresZ contains about 16 000 pre-processed SRA gene expression datasets of 23 different fungal species with industrial, agricultural and medical importance. Additional RNA-seq data will be updated periodically and upon request. FungiExpresZ’s intuitive user interface can also be used for the analysis of data from other organisms besides fungi.

## Materials and methods

### Design and implementation

FungiExpresZ was designed and implemented using R (version 3.6) and R-Shiny (version 1.3; https://cran.r-project.org/web/packages/shiny/). The R packages used in this work are listed in Supplementary Table 1. The web-based Shiny app can be accessed at: https://cparsania.shinyapps.io/FungiExpresZ/. The source code and detailed local installation instructions are available on GitHub (https://github.com/cparsania/FungiExpresZ).

### National center for biotechnology information (NCBI) sequence read archive (SRA) data pre-processing

At the time of publication, FungiExpresZ contains 15 954 processed RNA-seq datasets of 23 different fungal species. Except for *Saccharomyces cerevisiae,* raw reads (.fastq files) were downloaded from NCBI SRA and mapped against the respective reference genomes using HISAT2 [15] or TopHat2 [16] using the command shown in Supplementary Table 2. Only datasets with at least a 70% mapping rate to the respective reference genome are included in FungiExpresZ. Normalized gene expression values (expressed in Fragments per Kilobase of transcript per Million read pairs (FPKM)) were calculated for all genes in gene annotation files using Stringtie [17] or Cufflink [18] using the command listed in Supplementary Table 3. Reads mapping within the coordinates corresponding to the start and end of the ‘Gene’ feature in the gene annotation file were counted, normalized and expressed as FPKM. Reference genomes and gene annotation files can be found at https://github.com/cparsania/FungiExpresZ_reference_genomes. Information regarding the software tools and parameters used in data processing for each species is provided in Supplementary Tables 2 and 3, respectively. For *S. cerevisiae* data, normalized gene expression values (expressed in Tags Per Million (TPM)) were calculated by a previous study [4] using the STAR [19] and Kallisto [20] programmes. A table containing normalized gene expression values for all datasets can be downloaded from the ‘Downloads’ tab on the FungiExpresZ website (https://cparsania.shinyapps.io/FungiExpresZ/).

### Correlation, statistical and clustering analyses

Pair-wise correlation in scatter plots, multi-scatter plots and correlation heat-box plots are determined using Pearson’s correlation using R’s cor function. *P-*values are determined using Student’s *t*-test (t.test) or Wilcoxon signed-rank test (wilcox.test). Unsupervized clustering is performed using the *k*-means approach with all default parameters.

### Defining data groups

A tutorial video on sample and gene grouping is available at https://tinyurl.com/52pazteh. Details of how to incorporate sample and gene groups into plots can be found under the tab ‘About’ and ‘Step-by-step-tutorial’ on the FungiExpresZ page or from GitHub (https://tinyurl.com/56prkkya).

### PCA analysis and visualization

PCA analysis is implemented using the R function prcomp with default parameters. Users can define a subset of genes for PCA analysis, select principal components for visualization in a pair-wise scatter plot and colour by sample groups. A tutorial video is available at https://tinyurl.com/27bnurcc.

### Gene ontology analysis

Gene ontology (GO) enrichment analysis is performed using the enricher function from the clusterProfiler [21]. A full list of fungal species with species-specific GO annotations derived from FungiDB [14] can be found in Supplementary Table 4. The code to prepare the background data for GO analysis is provided on GitHub – https://tinyurl.com/26273dw3. Visualization of enriched GO terms, e.g. network diagram (CNET and EMAP plots), heatmap, bar plot, dot plot or UpSet plot, is supported by the enrichplot package [22].

### Text mining and word cloud

To generate a word cloud for selected SRA samples, redundant abstracts and common dictionary words are removed (Supplementary Table 5) before text mining and word frequency calculation with the TM [23]. Word cloud is generated using the R package wordcloud [24].

### Gene co-expression analysis

Gene co-expression analysis is based on clustering gene expression patterns of all genes or selected groups of genes. This can be done on FungiExpresZ using the clustering function on the public datasets. For the case of the SMURF (Secondary Metabolite Unique Regions Finder)-predicted sterigmatocystin (ST) genes [25], unsupervized *k*-means clustering was used to separate the genes into two clusters based on their expression patterns across 151 *Aspergillus nidulans* RNA-seq data available in FungiExpresZ. Briefly, the ST genes (Supplementary Table 6) were assigned as a group, and *k*-means clustering was performed on the *A. nidulans* datasets (*n* = 151) of different strains and growth conditions and visualized in a heatmap. To identify genes with a similar expression pattern to the ST genes, we assigned all genes other than the ST genes (*n* = 10 709) to another gene group. We then performed gene clustering based on an expression pattern similar to the ST genes (i.e. expressed in the same datasets). The number of *k*-means clusters for rows was empirically determined and set at five. GO enrichment analysis and visualization were performed using the GO enrichment analysis functionality on FungiExpresZ. Statistically significant (adjusted *P-*value < 0.01) GO terms were visualized in a network diagram (CNET plot).

## Results

### FungiExpresZ overview

FungiExpresZ offers an intuitive, user-friendly interface for analysing and visualizing RNA-seq data. Users can use the built-in plotting and analysis functions on their data alone or in combination with public RNA-seq datasets for integrated analyses. The output of each analysis can be directly visualized on the FungiExpresZ web interface or within the standalone tool. Currently, FungiExpresZ contains ~16 000 pre-processed RNA-seq datasets from 23 different fungal species (Table 1) that users can query to retrieve expression profiles of their genes of interest. The collection of public RNA-seq datasets includes wildtype and mutants of 23 different fungal species grown under various culture conditions (Supplementary Table 7) and will be updated periodically.

**Table 1:** List of species and number of SRA samples in FungiExpresZ

**Number**	**Species**	**Number of RNA-seq datasets**
1	*Aspergillus flavus*	348
2	*Aspergillus fumigatus*	242
3	*Aspergillus nidulans*	151
4	*Aspergillus niger*	253
5	*Beauveria bassiana*	226
6	*Botrytis cinerea*	142
7	*Candida albicans*	639
8	*Candida auris*	46
9	*Candida glabrata*	126
10	*Cordyceps militaris*	44
11	*Cryptococcus gattii*	11
12	*Cryptococcus neoformans*	385
13	*Fusarium fujikuroi*	74
14	*Fusarium graminearum*	344
15	*Fusarium oxysporum*	132
16	*Fusarium proliferatum*	4
17	*Fusarium verticillioides*	179
18	*Metarhizium anisopliae*	17
19	*Metarhizium robertsii*	79
20	*Saccharomyces cerevisiae*	11 872
21	*Pyricularia oryzae*	543
22	*Talaromyces marneffei*	26
23	*Ustilago maydis*	71
	Total	15 954

FungiExpresZ is an R-Shiny application (https://shiny.rstudio.com) that can be used online (https://cparsania.shinyapps.io/FungiExpresZ/) or runs as an R package on a local computer or server. A docker for one-click hassle-free standalone installation is available.

### FungiExpresZ data handling and integration

**Figure 1:**
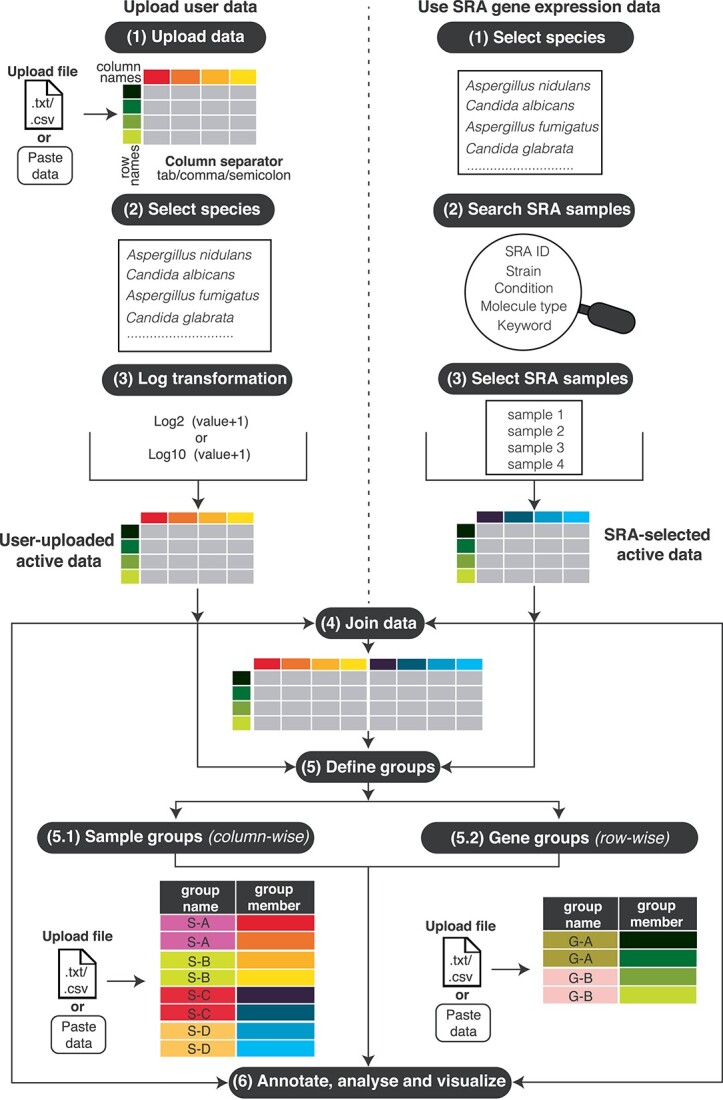
Schematic of the FungiExpresZ workflow. FungiExpresZ analysis of user data (Steps 1–3, left), SRA gene expression data (Steps 1–3, right) or both (Step 4). FungiExpresZ allows sample (i.e. datasets) and gene groupings (Step 5). Groups can be defined by supplying a text file or copying group information into the text area (Steps 5.1 and 5.2). Data annotation, analysis and visualization can be done on successfully uploaded data (Step 6).

An overall workflow of FungiExpresZ is summarized in Figure 1. The screenshot of the data upload page is shown in Supplementary Figure 1. User data are uploaded to FungiExpresZ using a text file or by pasting the data to the text entry box on the upload page (Figure 1 left panel, Step 1). Data can be in raw or normalized gene expression counts and need to be arranged in columns beginning with species’ gene names followed by numerical data (Supplementary Figure 2A) to utilize the graph plotting and data analysis functions. For comparisons with public data, data should be expressed as normalized gene expression counts (e.g. RPM, RPKM or FPKM). The header of each sample column is displayed as the data identifier in the drop-down menu of each analysis (Supplementary Figure 2B). As options, users can log-transform their data (log_2_ or log_10_) and select the species to be analysed (Figure 1 left panel, Steps 2 and 3). Users may use FungiExpresZ functions without species specification, but the gene description and GO functional annotation functionalities would not be supported.

User-uploaded data can be integrated with pre-processed public RNA-seq data using the ‘Join Data’ feature (Figure 1, Step 4). Datasets of interest can be searched using keywords such as species name, SRA ID, strain type and biological condition (Figure 1 right panel, Step 2) and can be assigned to user-specified sample groups (e.g. tissue type, growth media, biological or technical replicates) (Figure 1 Step 5, Supplementary Figure 3). Data analysis and visualization can be performed on user-uploaded and public data as a whole or on select samples and gene groups (Supplementary Figure 4).

### FungiExpresZ has many built-in visualization options

**Figure 2:**
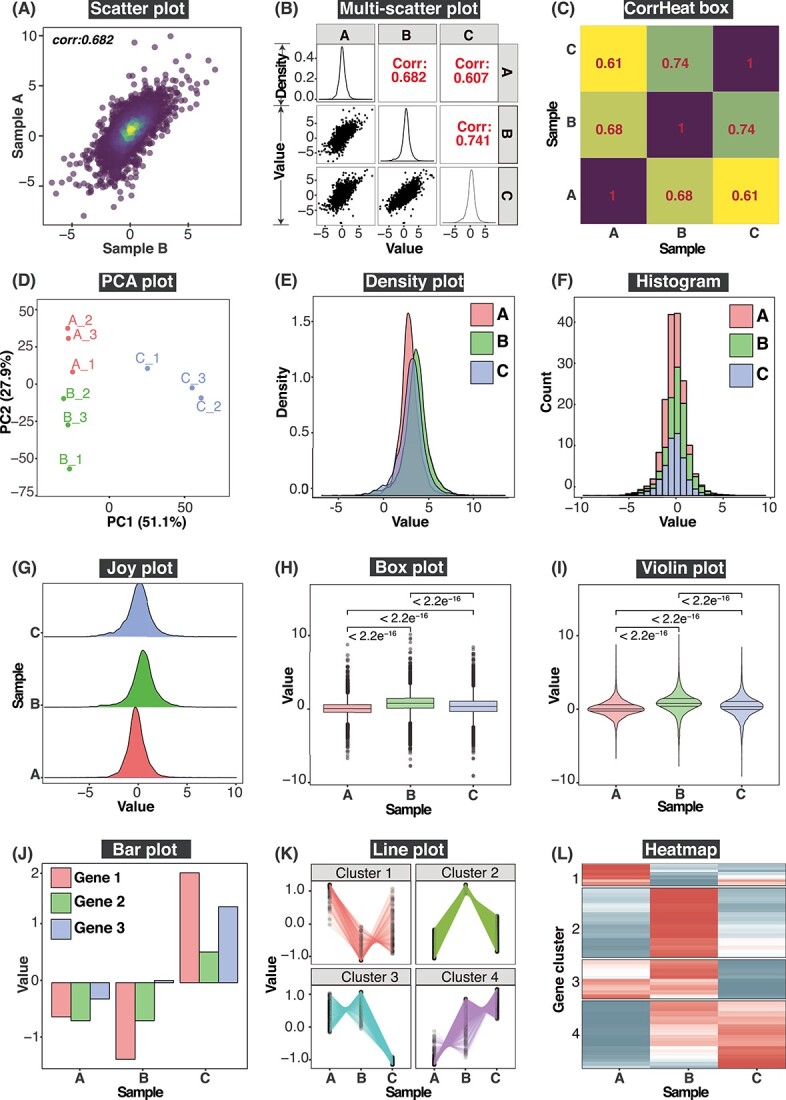
Exploratory data plots available in FungiExpresZ. FungiExpresZ offers 12 different highly customisable data exploratory plots: Scatter plot (**A**); Multi-scatter plot (**B**); CorrHeat box (**C**); PCA plot (**D**); Density plot (**E**); Histogram (**F**); Joy plot (**G**); Box plot (**H**); Violon plot (**I**); Bar plot (**J**); Line plot (**K**) and Heatmap (**L**).

FungiExpresZ offers many tools for data visualization, data quality evaluation and data comparison (Table 2). They include single and multiple sample-pair scatter plot, correlation heatmap, PCA, density plot, histogram, joy plot, box plot, violin plot, bar plot, line plot and heatmap (Figure 2A–L). The output graphs are highly customisable, allowing attributes such as the title, axis labels, axis tick labels, plot theme, legends, font size and resolution to be modified to yield publication-ready figures (Supplementary Figures 5 and 6). A video tutorial on creating the various plots is available at https://tinyurl.com/mtzhm86s.

**Table 2:** Types of analyses and visualizations provided in FungiExpresZ

**Type of analysis**	**Type of visualization**
Data quality check	Pair-wise scatter plot
	Multi-scatter plot
	Correlation heat box
	PCA plot
Data exploration	Density plot
	Histogram
	Joy plot
	Box plot
	Violin plot
	Bar plot
Clustering and pattern extraction	Heatmap
	Line plot
GO analysis and visualization	Dot plot
	Bar plot
	EMAP plot
	CNET plot
	UpSet plot
	Heatmap
Literature summary by text mining	Wordcloud

### FungiExpresZ offers many commonly used data analysis functions

**Figure 3:**
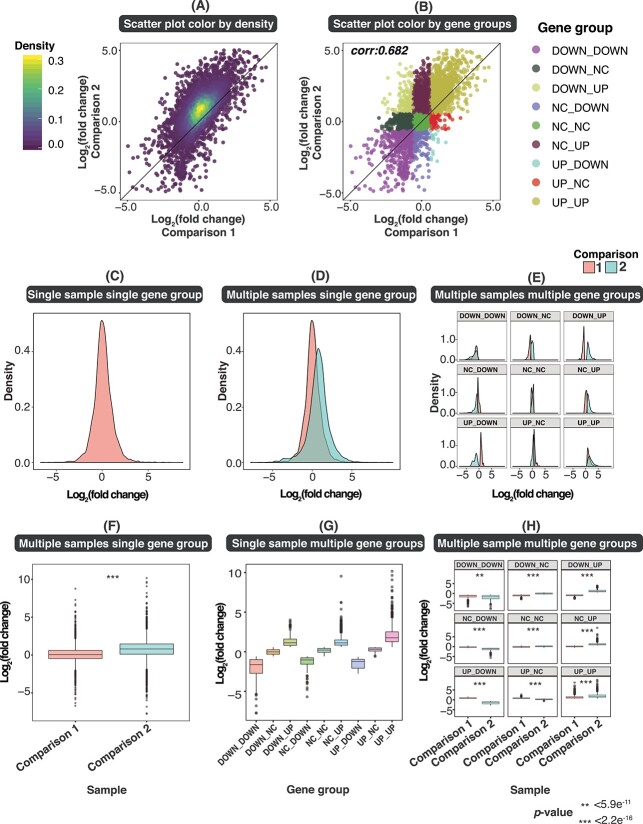
Visualization of grouped data in FungiExpresZ. FungiExpresZ offers several ways to visualize data grouped by genes or samples (i.e. datasets). Gene or sample groups can be plotted separately and coloured differently. Data of all genes (**A**, **C** and **F**) or user-defined groups of genes (**B**, **E** and **H**) from a single dataset (**C** and **G**) or multiple datasets (**A**, **B**, **D**, **E**, **F** and **H**) can be plotted and visualized on the same graph.

**Figure 4:**
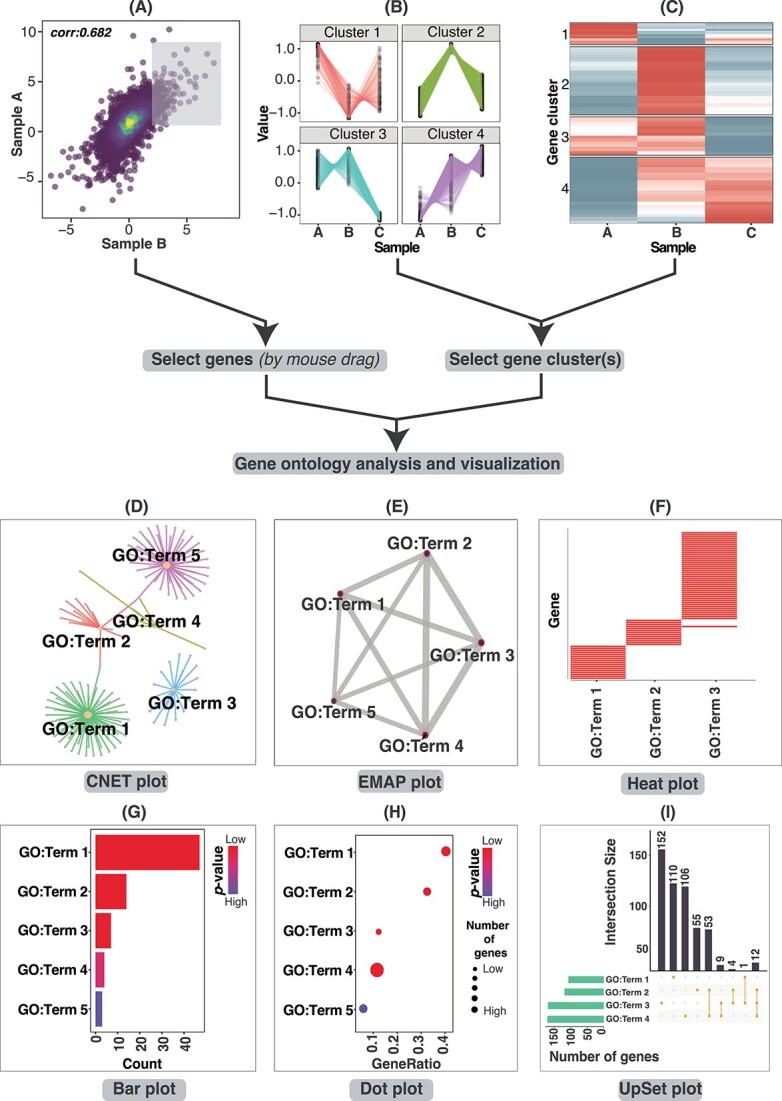
GO analysis and visualization in FungiExpresZ. FungiExpresZ can subject selected gene sets from the scatter plot (**A**), line plot (**B**) and heatmap (**C**) to GO analysis and visualization. Enriched GO terms can be visualized in six different types of plots—CNET (**D**), EMAP (**E**), Heatmap (**F**), Bar (**G**), Dot (**H**) and UpSet (**I**) plots.

Clustering is a powerful standard method for gene expression analysis [26]. Two unsupervized clustering methods are supported by FungiExpresZ—namely *k*-means and hierarchical clustering. Data points of different gene clusters or user-defined gene groups can be colour-coded to better present multi-dimensional expression data (Figure 3 and Supplementary Figure 7). Moreover, gene description and gene expression values of selected genes or gene clusters in scattered plots, line plots and heatmaps can be obtained from the summary table at the bottom of the page (Supplementary Figure 8). FungiExpresZ also offers GO functional enrichment analysis [27, 28] for 126 fungal species (Supplementary Table 4). GO analysis results can be downloaded (as .xlsx, .csv or .pdf files) (Supplementary Figure 9) and visualized in six ways: network diagrams (CNET and EMAP plots), heatmaps, bar graphs, dot plots and UpSet plots (Figure 4). Users can determine the number of significant GO terms to be displayed.

Gene co-expression analysis can easily be performed on FungiExpresZ using the *k*-means clustering function. After uploading data and/or retrieving public data for a given organism, users can choose to perform co-expression analysis (i.e. clustering) on all genes, a given number of genes with the highest or lowest expression or a user-defined gene list.

FungiExpresZ also contains a text-mining function (Supplementary Figure 10) for acquiring information from the description of selected datasets and the abstract of papers associated with selected datasets. This function aims to provide a quick overview of the biological relevance of selected datasets based on keywords (e.g. gene names, cellular function, experimental condition) enriched in the experimental descriptions and abstracts presented in a word cloud graphic.

### Cases illustrating applications of the public datasets and analysis tools in FungiExpresZ

#### Case 1: Identifying secondary metabolism gene cluster boundaries

Fungi produce many chemical compounds through highly specialized metabolic pathways, generally called secondary metabolism (SM). Genes involved in a given SM pathway are usually arranged in a cluster in fungal genomes [29]. Bioinformatics tools have been developed to identify SM clusters from genome sequences. One of the challenges for SM cluster annotation is defining the cluster boundaries. Given that SM cluster genes are silent under most conditions and co-regulated by a transcription factor that usually resides within the cluster [29], co-expression analysis can be a powerful way to define cluster genes and boundaries.

**Figure 5:**
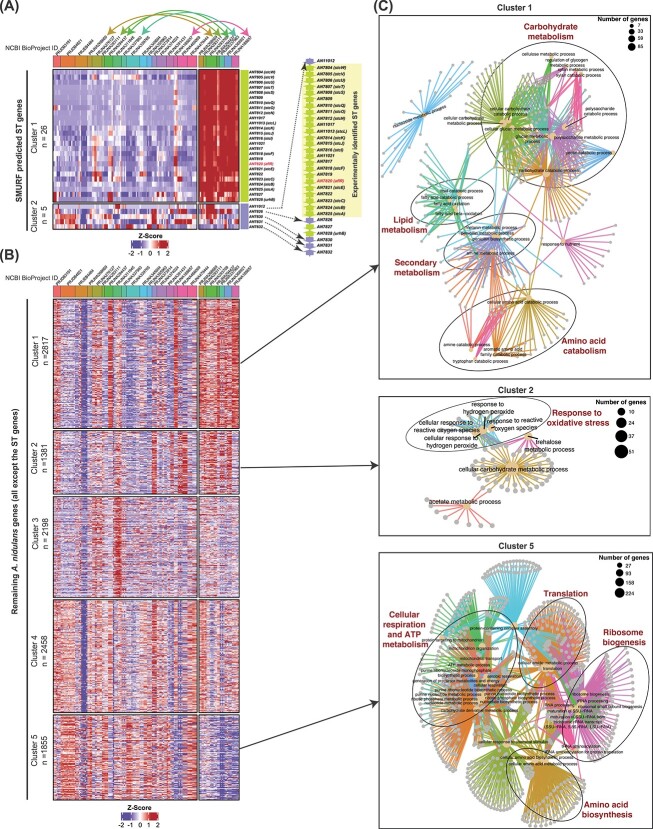
Examples of co-expression analysis using public RNA-seq data available in FungiExpresZ. A heatmap diagram showing the expression pattern (presented as z-scores) of the ST gene cluster predicted by SMURF across 151 public RNA-seq data of *A. nidulans* in FungiExpresZ (**A**). Genes (rows) and datasets (columns) were clustered based on the expression patterns of the SMURF-predicted ST genes using *k*-means clustering. Columns (datasets) are annotated with NCBI Bio-Project ID, which is also depicted by unique colour-coded bars. Colour-coded arrows highlight datasets from the same Bio-Project ID but in separate column clusters. The schematic diagram on the right of the heatmap presents the gene arrangement of the ST cluster. Arrowheads indicate gene directions. Gene clusters (row clusters) and the corresponding ST genes on the gene arrangement schematic diagram are colour-coded. A heatmap diagram shows the expression pattern (presented by z-score) of all *A. nidulans* genes except for the ST genes (**B**). All *A. nidulans* genes (except the ST genes) were clustered by gene expression using *k*-means clustering. Columns (datasets) are arranged in the same order as those for the ST genes in (**A**). Columns (datasets) are annotated with NCBI Bio-Project ID, which is also depicted by unique colour-coded bars. Clusters 1, 2 and 5 genes from (**B**) were selected for GO enrichment analysis. Enriched GO terms are displayed in CNET plots (**C**). Data shown in this figure are provided in Supplementary Tables 10–15.

To demonstrate this, we performed *k*-means clustering on the expression of genes predicted by SMURF [25] for the well-established sterigmatocystin (ST) cluster of *A. nidulans* from published RNA-seq datasets (*n* = 151). Two clusters with distinct gene expression patterns were observed (Figure 5A). Cluster 1 contains tightly co-regulated genes (e.g. commonly induced in 33 datasets from different projects), including all the genes that were experimentally shown to be involved in ST biosynthesis [30]. On the other hand, cluster 2 has five genes with a different expression pattern (e.g. not expressed in most of the 33 datasets), suggesting that they are dispensable for ST biosynthesis and, therefore, not belonging to the ST gene cluster. Consistent with this, the five genes have been experimentally excluded from the defined ST gene cluster [30] (Figure 5A, right panel). This example illustrates the usefulness of FungiExpresZ-based co-expression analysis of public data for predicting SM cluster genes in fungi.

#### Case 2: Uncovering genes and physiological pathways associated with SM production

Co-expression analysis can also be used to gain insights into the metabolism and physiological processes associated with the production of a given SM. For example, we performed *k*-means clustering on all *A. nidulans* genes to identify genes with a similar expression pattern as the ST cluster genes. Two clusters of genes (clusters 1 and 2) have relatively higher expression in those datasets (*n* = 33) in which ST genes were induced (Figure 5B). GO enrichment analysis showed that these genes are enriched in functions related to amino acid catabolism, carbohydrate, lipid and SM and oxidative stress response (Figure 5C), suggesting that these physiological and metabolic functions are associated with ST production in *A. nidulans.* On the other hand, cluster 5 genes have an expression pattern opposite to clusters 1 and 2 genes and the ST genes. These genes are expressed when ST genes are silent, and vice versa (Figure 5B). They are enriched with functions related to respiration, adenosine triphosphate and amino acids metabolism, translation and ribosome biogenesis (Figure 5C), processes required for primary metabolism and growth but dispensable during the stationary growth phase when SM occurs.

#### Case 3: Revealing the activity and potential functions of transcription factors

**Figure 6:**
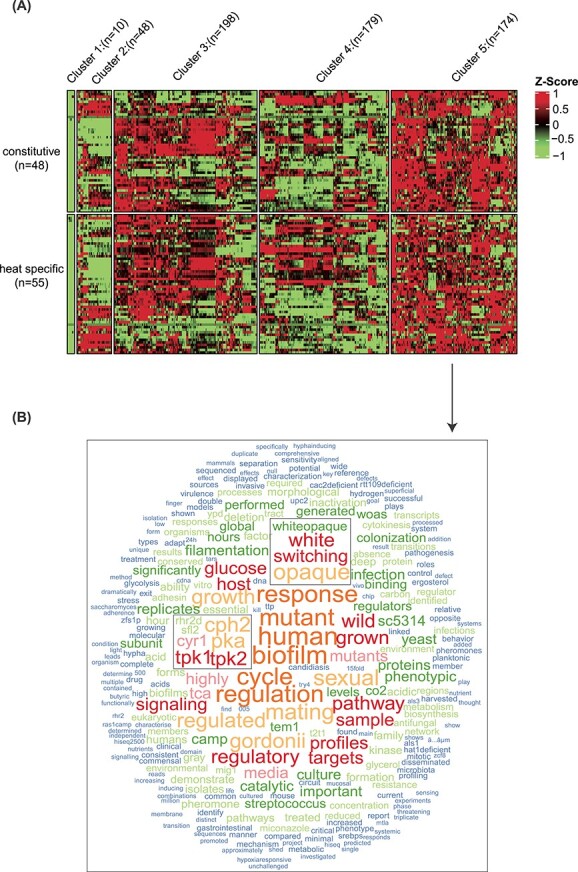
Demonstration of word cloud utility in FungiExpresZ. The 609 public RNA-seq datasets of *C. albicans* were clustered (by columns) based on the expression patterns of Hsf1 target genes in FungiExpresZ (**A**). The descriptions of datasets and studies in Cluster 5 (*n* = 174) were subjected to the text-mining function in FungiExpresZ. The result is presented in the word cloud plot (**B**). The square boxes in (**B**) highlight the physiological processes and genes previously shown to be associated with heat and Hsf1 in *C. albicans*.

Transcription factors’ activity can be inferred from the expression patterns of their target genes, which can be determined easily from the public data available in FungiExpresZ. We demonstrate this on the conserved heat shock factor Hsf1 of *Candida albicans*, the function of which is regulated by temperature and essential for transcriptional activation of heat shock response genes [31, 32]. We interrogated 609 public *C. albicans* RNA-seq datasets in FungiExpresZ for conditions that induce the expression of Hsf1 direct gene targets (*n* = 104) [32]. The Clustering analysis identified 174 datasets from 29 separate studies (Supplementary Table 8) showing high expression levels for the Hsf1 targets (Figure 6A, cluster 5). To gain insight into what conditions Hsf1 is active, the abstracts of those datasets were selected for text mining using the Word Clouds function in FungiExpresZ. The computed word cloud shows enriched keywords such as ‘white opaque’, ‘white’, ‘opaque’ and ‘switching’ (Figure 6B). This is in line with previous findings suggesting that temperature is associated with changes between the two physiological forms of *C. albicans* white and opaque cells [33–35]. In addition, there are several proteins (e.g. Cph2, Pka, Tpk1, Tpk2, Cyr1, Rhr2d and Sfl2) enriched in the description of the datasets with some of the proteins previously reported to have a functional association with temperature or being regulated by temperature [36–39]. This example illustrates how FungiExpresZ can be used to obtain quick, meaningful insights into potential biological connections among select genes from published datasets and their associated literature.

#### Case 4: Identifying candidate transcription factors for a group of co-regulated genes

Based on the same principles applied in Case 3, the public data of FungiExpresZ can also be used to identify candidate transcription factor(s) for a given set of co-regulated genes, such as those involved in a specific physiological process or metabolic pathway [40, 41]. Co-expressed transcription factor genes can be determined using the *k*-means clustering function on FungiExpresZ to group transcription factor genes with the genes of interest based on their expression patterns in public datasets.

### FungiExpresZ documentation and support

To demonstrate the various utilities in FungiExpresZ, we have included an example analysis of previously published RNA-seq data [32]. The example can be found under the ‘About → Overview’ tab. In addition, a step-by-step user guide is provided under the ‘About → Step-by-step tutorial’ tab and videos for various FungiExpresZ utilities can be found at https://tinyurl.com/mtzhm86s (Supplementary Table 9). Users can submit questions or report bugs on the FungiExpresZ support page at GitHub (https://github.com/cparsania/FungiExpresZ/issues).

### FungiExpresZ limitations

The current version of FungiExpresZ has a few limitations. The online version of FungiExpresZ has a default 30 min idle-time limit, after which the session would be suspended. Analysis and visualization made in expired sessions are not saved, and users need to initiate a new session and re-upload data to continue analysis. A user login feature may be introduced as an option in a future update to allow the data-saving function. On the other hand, there is no time limit if FungiExpresZ is installed on a local computer or network with the uploaded data saved until the FungiExpresZ webpage is closed. Therefore, the online version is useful for quick and straightforward data exploration and analysis. The locally installed version is ideal for analyses involving large numbers of datasets or datasets of other fungal species and organisms not available in the FungiExpresZ collection. Another limitation of FungiExpresZ is the lack of a tool for differential expression analysis of user-supplied or public data. This function will be added along with other tools (e.g. Kyoto Encyclopedia of Genes and Genomes (KEGG) pathway analysis, DNA binding motif enrichment analysis and orthologous gene expression analysis) in future updates to expand on the functionality of FungiExpresZ.

## Discussion

RNA-seq has become a standard and indispensable approach in molecular biology research [42]. There is an ever-increasing demand for bioinformatics expertise to carry out data analysis. FungiExpresZ offers an easy integrative platform to perform standard data analysis and generate publication-quality figures through only a few clicks, overcoming the major limitation in data analysis faced by many wet-lab scientists. Although primarily targeting scientists with little or no bioinformatics experience, FungiExpresZ offers many optional parameters in each function for advanced analyses. Therefore, it is also suitable for experienced bioinformaticians, who may take advantage of the platform for quick data visualization and analysis before embarking on specialized analyses. In addition, the intuitive nature of FungiExpresZ makes it ideal for educational purposes, e.g. training bioinformatics novices on the application of the different analysis methods without bothering with complicated programming scripts. FungiExpresZ comes in both web-based and standalone versions, with the latter allowing offline analysis and the incorporation of additional datasets (e.g. lab-generated published data or data of other organisms not included in the FungiExpresZ collection) to the existing collection. Therefore, FungiExpresZ is suitable for users of different bioinformatics capabilities.

The number of publicly available RNA-seq data has increased exponentially [42] with large amounts of raw datasets deposited daily to public domains. However, a uniform processing pipeline is needed for reliable comparisons between datasets generated from different studies [4] and processing large amounts of data can be time-consuming and computationally demanding. As such, the lack of uniformly processed public data is a bottleneck to harnessing the potential of the rich public data. FungiExpresZ also caters to this problem for the fungal field by offering easy access to an extensive collection of pre-processed public RNA-seq datasets of many fungal species and gene and GO information of more than 120 fungal species. New gene expression data and information will be routinely updated as they become available or upon request.

As genomics and bioinformatics have become a standard approach nowadays for probably all organisms, it is expected that bioinformatics analysis tools like FungiExpresZ will be in increasing demand. The FungiExpresZ platform developed in this work can be easily extended to support any organism of interest, and the development of a similar platform for other organisms is underway.

**Key Points**

- FungiExpresZ is an intuitive and user-friendly bioinformatics tool for data analysis and a database of fungal gene expression profiling data.
- FungiExpresZ is built with wet-lab scientists in mind to overcome their computational and bioinformatic limitations in data analysis.
- FungiExpresZ offers 19 different bioinformatics analyses, and their outputs are provided in publication-ready figures.
- FungiExpresZ is a valuable resource for the research community, particularly the fungal field, as it contains more than 16 000 pre-processed RNA-seq data of many fungal species, including model fungi and human, plant and insect pathogens.

## Supplementary Material

FungiExpresZ_supp_figures_1_bbad051

FungiExpresZ_supp_figures_2_bbad051

FungiExpresZ_supp_figures_3_bbad051

FungiExpresZ_supp_figures_4_bbad051

FungiExpresZ_supp_figures_5_bbad051

FungiExpresZ_supp_figures_6_bbad051

FungiExpresZ_supp_figures_7_bbad051

FungiExpresZ_supp_figures_8_bbad051

FungiExpresZ_supp_figures_9_bbad051

FungiExpresZ_supp_figures_10_bbad051

FungiExpresZ_supp_tables_bbad051

Supplementary_Figure_legends_bbad051

## Data Availability

FungiExpresZ web server can be accessed online at https://cparsania.shinyapps.io/FungiExpresZ/, while the source codes and the instructions for local installation are available on GitHub (https://github.com/cparsania/FungiExpresZ). Preprocessed public RNA-seq data can be found under the ``Download'' tab on the FungiExpresZ webpage (https://cparsania.shinyapps.io/FungiExpresZ/) or at https://github.com/cparsania/FungiExpresZ/tree/master/inst/app/expression_mats_rds_files_new.

## Author Biographies

**Chirag Parsania** was a PhD student in the Faculty of Health Sciences, University of Macau, Macau SAR of China, and is now a postdoctoral fellow at the Centenary Institute, Australia.

**Ruiwen Chen** is a master’s student in the Faculty of Health Sciences, University of Macau, Macau SAR of China.

**Pooja Sethiya** was a PhD student in the Faculty of Health Sciences, University of Macau, and is now a postdoctoral fellow at the Westmead Institute for Medical Research, Australia.

**Zhengqiang Miao** is a PhD student in the Faculty of Health Sciences, University of Macau, Macau SAR of China.

**Liguo Dong** was a PhD student in the Faculty of Health Sciences, University of Macau, Macau SAR of China.

**Koon Ho Wong** is an Associate Professor in the Faculty of Health Sciences, University of Macau, Macau SAR of China.
