# Supplementary figures and images for: FungiExpresZ: an intuitive package for fungal gene expression data analysis, visualization and discovery
Source: Brief Bioinform. 2023 Feb 17;24(2):bbad051. doi: 10.1093/bib/bbad051 (PMC10025439; doi:10.1093/bib/bbad051)

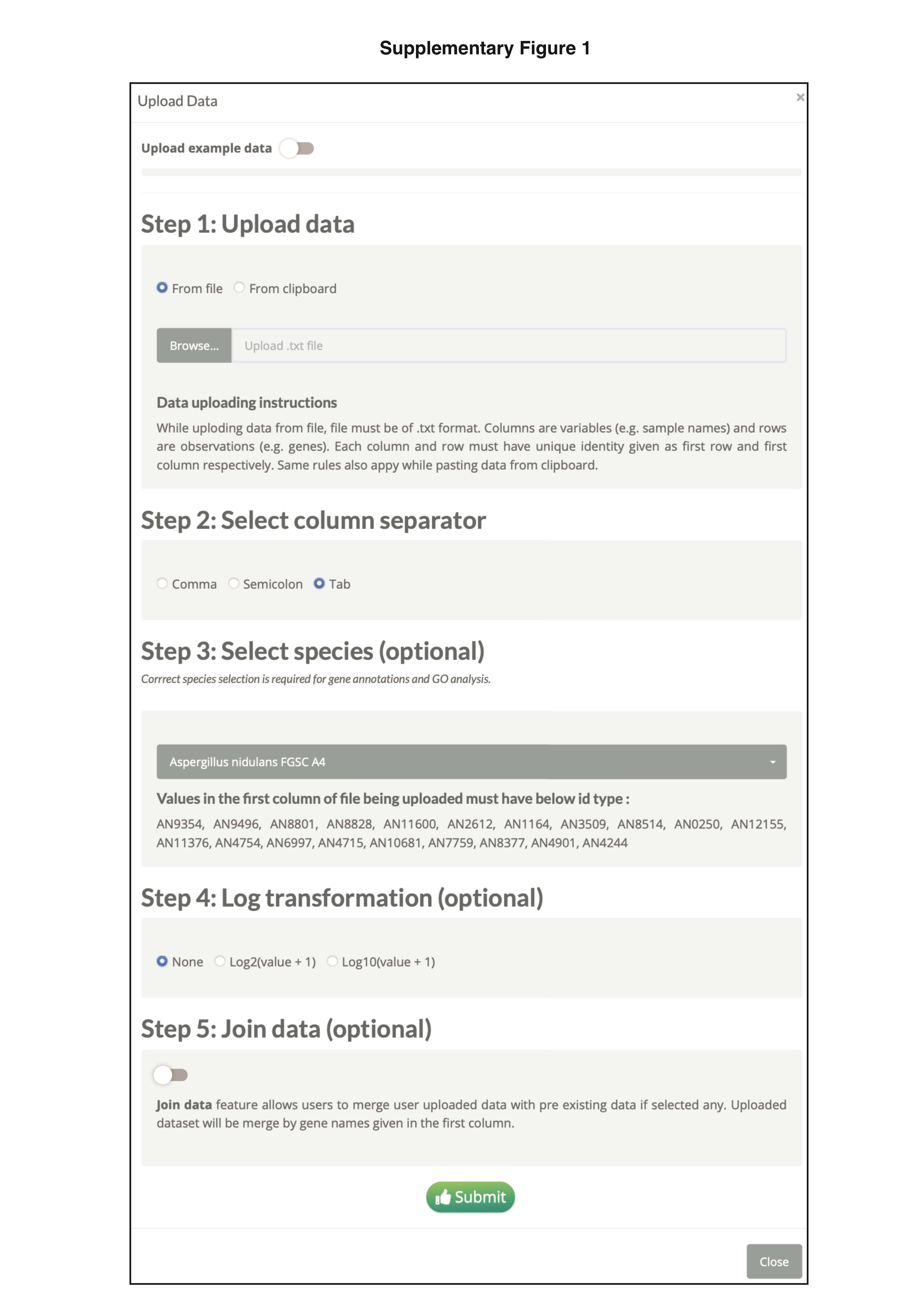

Supplement: FungiExpresZ_supp_figures_1_bbad051 [file fungiexpresz_supp_figures_1_bbad051.jpeg]

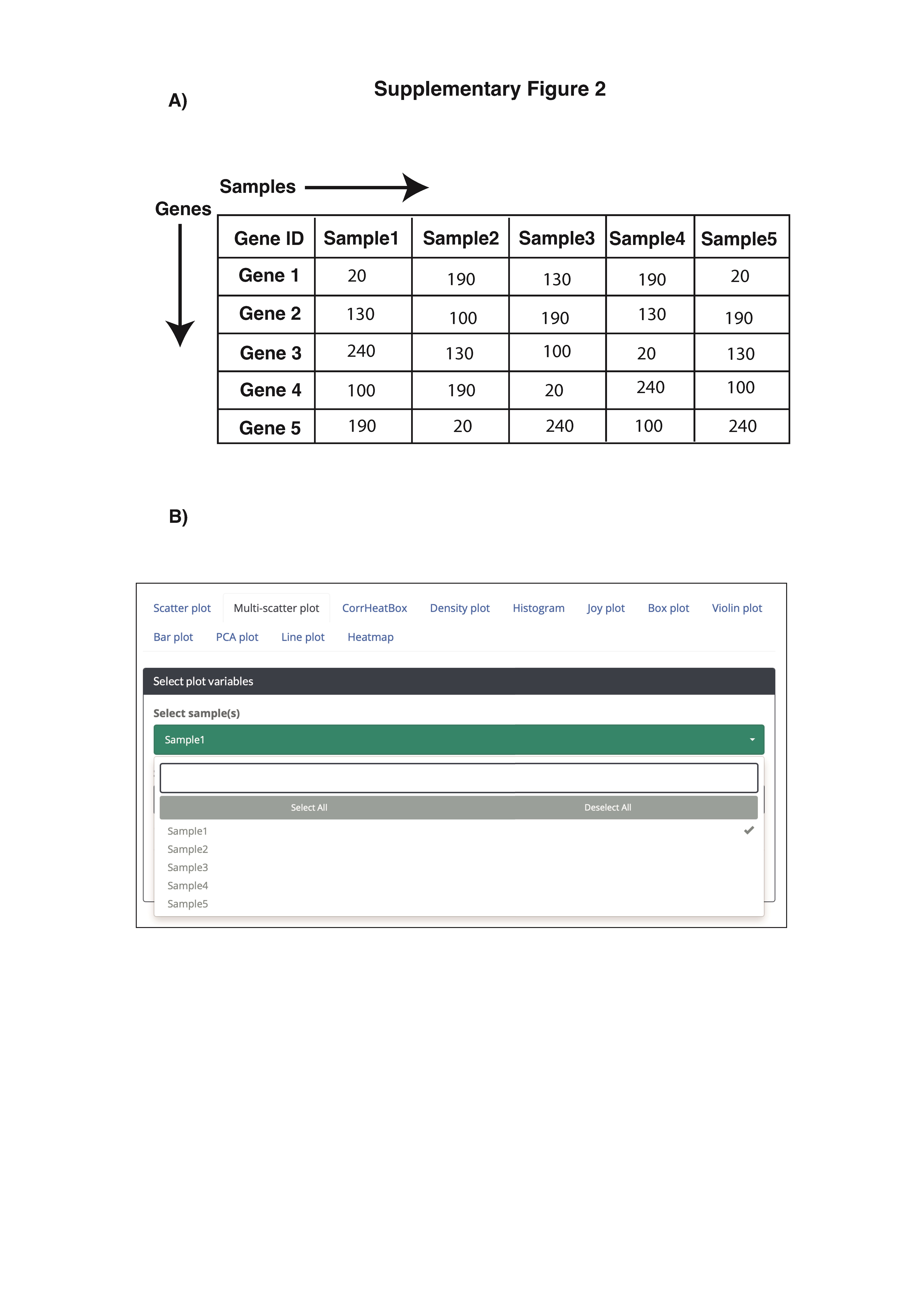

Supplement: FungiExpresZ_supp_figures_2_bbad051 [file fungiexpresz_supp_figures_2_bbad051.jpeg]

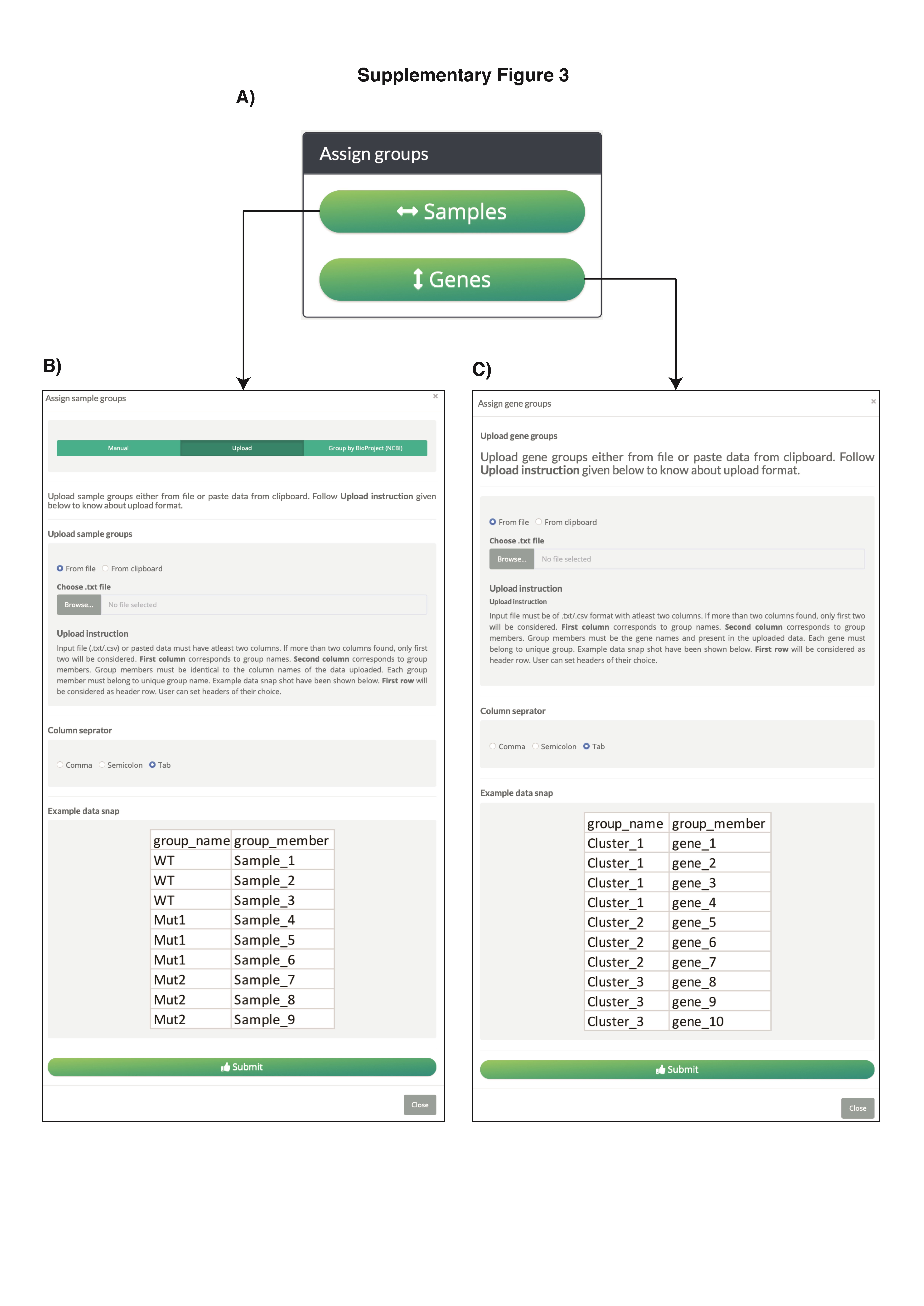

Supplement: FungiExpresZ_supp_figures_3_bbad051 [file fungiexpresz_supp_figures_3_bbad051.jpeg]

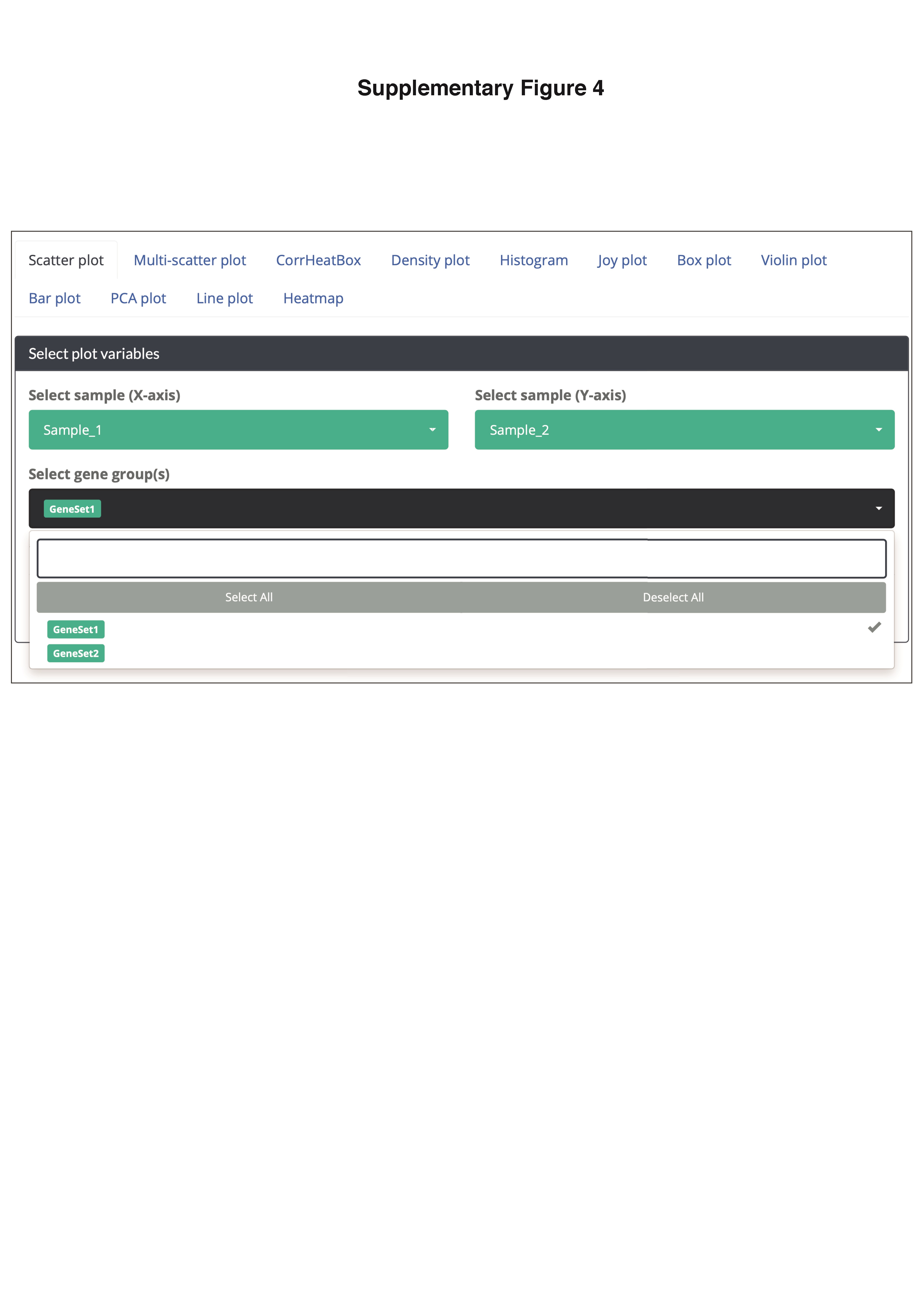

Supplement: FungiExpresZ_supp_figures_4_bbad051 [file fungiexpresz_supp_figures_4_bbad051.jpeg]

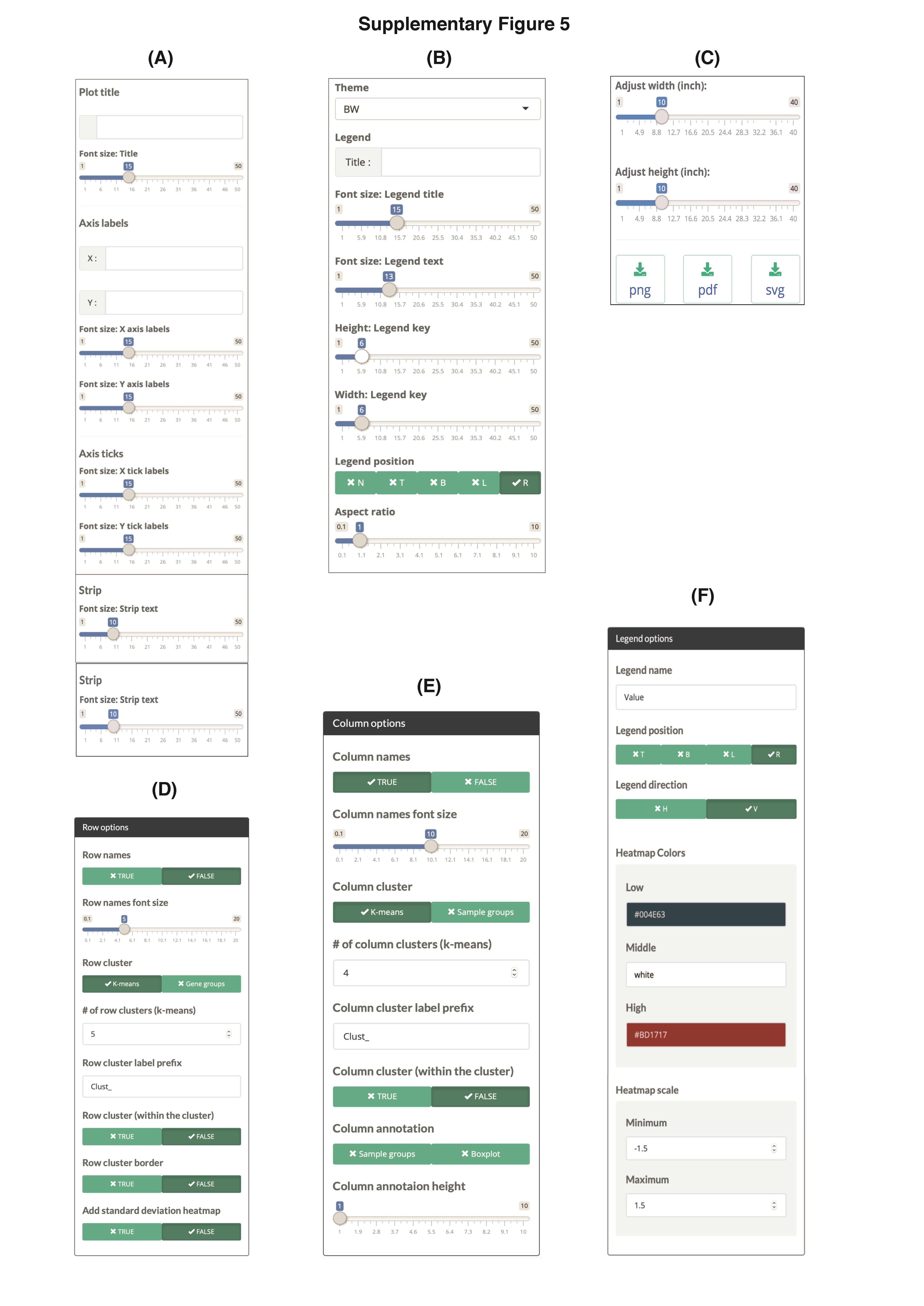

Supplement: FungiExpresZ_supp_figures_5_bbad051 [file fungiexpresz_supp_figures_5_bbad051.jpeg]

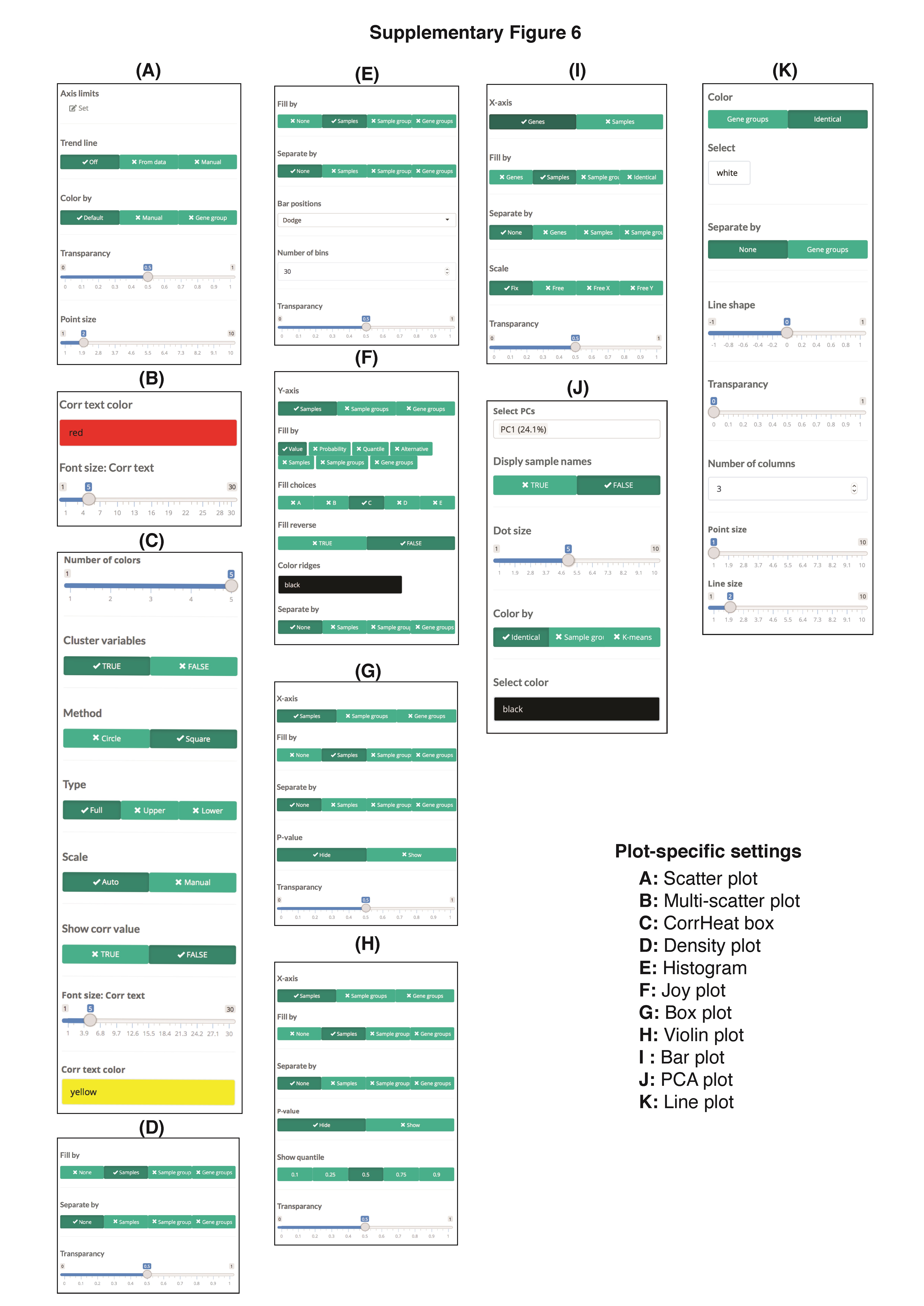

Supplement: FungiExpresZ_supp_figures_6_bbad051 [file fungiexpresz_supp_figures_6_bbad051.jpeg]

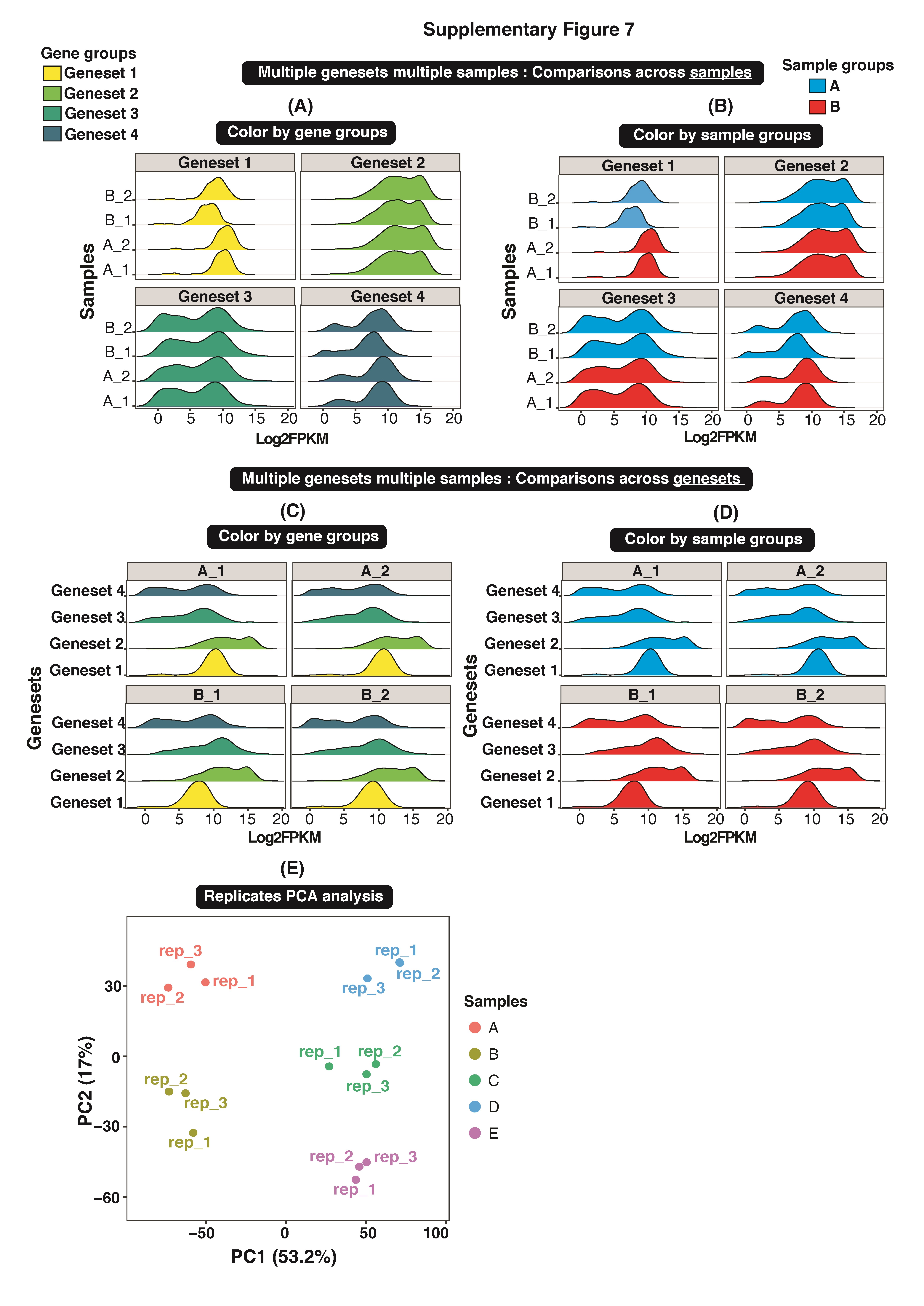

Supplement: FungiExpresZ_supp_figures_7_bbad051 [file fungiexpresz_supp_figures_7_bbad051.jpeg]

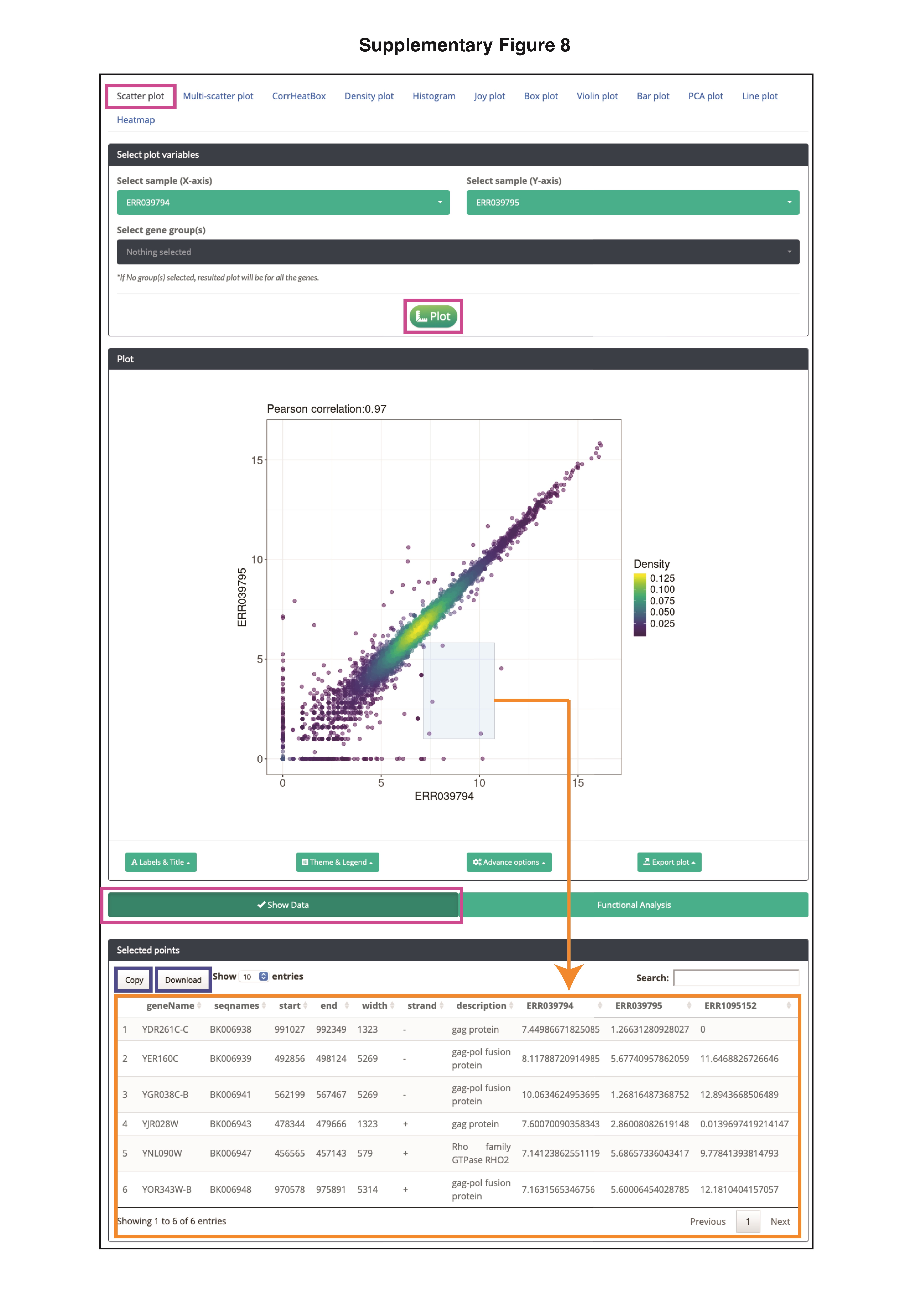

Supplement: FungiExpresZ_supp_figures_8_bbad051 [file fungiexpresz_supp_figures_8_bbad051.jpeg]

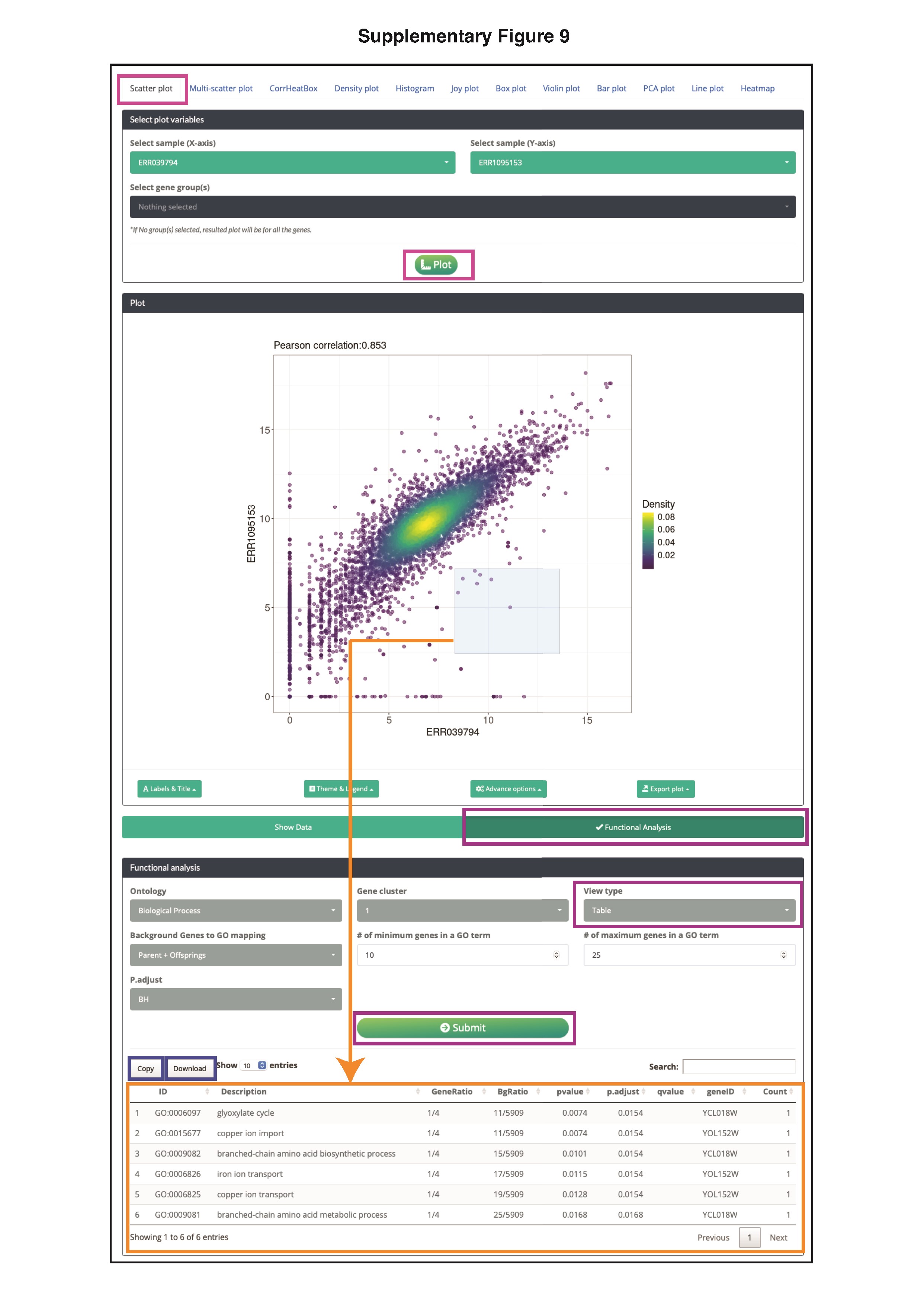

Supplement: FungiExpresZ_supp_figures_9_bbad051 [file fungiexpresz_supp_figures_9_bbad051.jpeg]

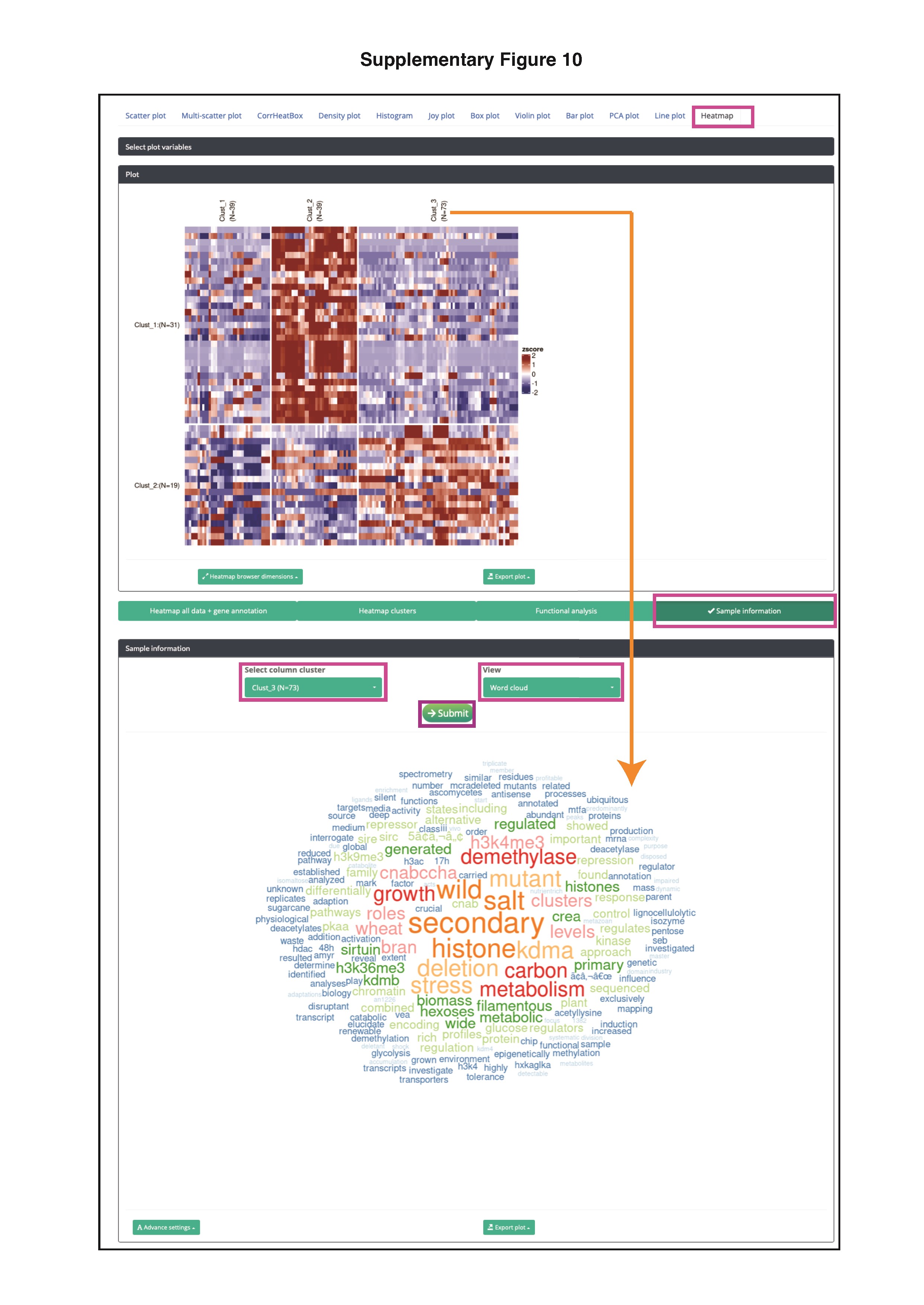

Supplement: FungiExpresZ_supp_figures_10_bbad051 [file fungiexpresz_supp_figures_10_bbad051.jpeg]
