# Supplementary material for: FungiExpresZ: an intuitive package for fungal gene expression data analysis, visualization and discovery
Source: Brief Bioinform. 2023 Feb 17;24(2):bbad051. doi: 10.1093/bib/bbad051 (PMC10025439; doi:10.1093/bib/bbad051)
Supplement: Supplementary_Figure_legends_bbad051 [file supplementary_figure_legends_bbad051.docx]

**Supplementary Figure 1. A screenshot showing the user data upload panel in FungiExpresZ**

Users can upload data using a .txt file or paste it to the clipboard (Step 1). Users need to define the column delimiter (e.g. comma, a semicolon, or a tab) used in the data format (Step 2). Users can select the species to which the data belongs (Step 3). Species selection is optional but allows users to obtain gene descriptions and perform gene ontology analysis. As an option, data can be transformed into log_2_ or log_10_ values at the upload step (Step 4). Finally, users have a choice to join uploaded data with the pre-processed data in FungiExpresZ using the “join data” button (Step 5).

**Supplementary Figure 2. Data format and upload**

FungiExpresZ allows users to upload data in a tabular format. Gene and dataset information are arranged in rows and columns, respectively (A). The first row and first column denote row and column names, respectively. Columns must be delimited by a comma, a semicolon, or a tab. Once the data is uploaded, column names will appear in the sample selection drop-down menu of the various plot panels (B).

**Supplementary Figure 3. A screenshot showing how to add gene and sample grouping information in FungiExpresZ**

FungiExpresZ allows the analysis and visualisation of grouped data. Groups can be defined by samples (e.g. user-defined datasets or public SRA datasets) and/or genes (A). Gene and sample grouping information can be input by uploading a file or pasting it on the clipboard text box (B and C). The data format for the grouping information is shown.

**Supplementary Figure 4. A screenshot showing how to select gene groups for analyses in FungiExpresZ**

In any given plotting function, users have a choice to visualise the data of the user-defined group(s) of genes from all datasets or the user-defined datasets by selecting the gene and sample groups from the drop-down menu, as shown in the figure.

**Supplementary Figure 5. Screenshots showing plot setting options in FungiExpresZ**

FungiExpresZ allows modifications of plot attributes such as plot title, axis labels, font size, legend title, legend position, legend size, plot width, plot height and plot export. These settings are grouped into three categories: Labels & Titles (A); Theme & Legend (B); and, Export plot (C). Heatmap has additional settings for rows (D), columns (E), and legends (F).

**Supplementary Figure 6. Screenshots showing plot-specific settings in FungiExpresZ**

FungiExpresZ provides many plot-specific options. Scatter plot (A) has options to set axes limits, display trend lines, colour gene groups and set colour transparency and data point size. Multi-scatter plot (B) has options to set font colour and size. CorrHeat box (C) has options to choose the number of colours in the scale bar, perform data clustering, choose plot appearance (e.g. square or circle value boxes, full or half plots), and set font size and colour. Density plot (D) has options to set the colour and transparency for data points that can be grouped and displayed by samples, sample groups or gene groups. Histogram (E) has options to set the colour and transparency for data points that can be grouped and displayed by samples, sample groups or gene groups and adjust the position of bars and the number of bins to display. Joy plot (F) has options to group data on the y-axis by samples, sample groups, or gene groups. Data points can be coloured by raw values, probabilities, quantiles, samples, sample groups, or gene groups. Users can choose the colour for the ridges for different samples, sample groups or gene groups. Box (G) and plots (H) have options to group and colour data by samples, sample groups, or gene groups. Users can choose to display or hide the *p-*value between each sample pair and set the colour transparency of data boxes. Violin plot (H) also has the option to display multiple quantile values. Bar plot (I) has the option to group data by genes or samples. Datapoints can be coloured separately by genes, samples, or sample groups. Users can choose the appearance of data bars (e.g. same or different colours and transparency) and set axes scales. PCA plot (J) has options to display or hide sample names, change their font size, and adjust datapoint colour and size. Line plot (K) has options to display lines for individual genes or average expression of all or selected groups of genes, set the colour and size of lines and data points, and adjust the number of columns to display.

**Supplementary Figure 7. Visualisation of grouped data in FungiExpresZ**

FungiExpresZ can visualise data grouped by genes and/or samples (e.g. datasets). Groups can be plotted and coloured differently on the joy (A to D) and PCA (E) plots.

**Supplementary Figure 8. A screenshot showing a summary table of the genes selected from a scatter plot.**

FungiExpresZ provides gene information (e.g. genomic co-ordinates, function description and gene expression values across user-uploaded and public datasets) for selected gene(s) on the scatter plot. Genes on the scatter plot can be selected by mouse drag (the blue box in the middle panel). Summary of each gene will be displayed in a table (bottom panel) which can be copied to clipboard or downloaded in .xls, .csv or .pdf formats. The relevant buttons to click for displaying the summary table are boxed.

**Supplementary Figure 9. A screenshot showing the output of GO analysis on genes selected from a scatter plot.**

FungiExresZ allows GO analysis to be performed on genes selected from the scatter plot (top panel) through mouse drag (middle panel). GO analysis output can be displayed in a table (bottom panel), copied to clipboard and downloaded in .xls, .csv or .pdf formats. The relevant buttons to click for performing GO analysis are boxed.

**Supplementary Figure 10. A screenshot showing a wordcloud functionality implemented in FungiExpresZ.**

FungiExpresZ provides the wordcloud functionality accessible through the clicking “Heatmap” ↓ “Plot” ↓ “Sample information” buttons highlighted in the figure. The relevant buttons to click for applying the Wordcloud function are boxed.
